# Habit Spasms

**Published:** 1895-11

**Authors:** E. T. Loeffler

**Affiliations:** Saginaw, Mich.


ARTICLE VI.

### HABIT SPASMS.

BY DR. E. T. LOEFFLER, SAGINAW, MICH.

Children often, and adults sometimes, present spasmodic movements, such as winding, twitching the mouth, jerking the head, movements that have a half-voluntary aspect, but which the individuals are unable to control. The patient is said to have "got a trick" of moving the part. Weir Mitchell calls this "habit chorea," but "habit spasm" is perhaps the better term.

The condition is met with chiefly in childhood, especially in the second half, but it sometimes commences in youth, and even in adult life. In young women it is often associated with symptoms of hysteria, and there may be a difficulty in deciding whether certain spasmodic movements are to be regarded as of reflex dental neurosis, of habit spasm or of hysterical spasm.

When it commences in childhood, the affection commonly ceases after a few months or a year, but it occasionally goes on to middle life or even longer. Rarely it begins late in life, but is then generally permanent. In early life it occurs especially in nervous and excitable children. The affection is said to be more common in females than in males, but is often seen in boys. Some writers claim that impairment of general health often precedes the development of the movements; occasionally they appear to be due to some special influence depressing the nervous system, overwork at school, a fright or some injury. In one case, for instance, the onset followed a fall into water.

Again we notice that frequently there is a history of other neurosis in parents or other relatives. It is quite probable that the affection often arises by imitation; not direct imitation, perhaps, but the witnessing of such movements is apt to produce a peculiar excitability, which finds expression and relief in movements of a similar nature. In cases in which something like direct inheritance can be traced, it is probable that this influence has been at work. A father, for instance, had such movements in the face all through his life, and two children likewise presented them. So we must frankly admit that in many cases no causes can be traced, and the affection seems to be the result of the restlessness of childhood, specialized, as it were, in a particular direction. In one instance a relative of mine, a girl, who began to blink the eyes in early childhood, still did so at the age of thirty.

In another case reported to me, a clergyman of thirty-seven was greatly annoyed by an involuntary smile, of somewhat meaningless aspect, which would cross his face from time to time without the slightest corresponding emotion, and even when he was engaged in public in the most solemn parts of the church service. It never troubled him when he was preaching or in conversation, but it often occurred when he was looking at another person and sometimes gave rise to misconception. It commenced at the age of sixteen, and was at first more than a smile, being actual laughter, but it gradually subsided to its present form.

The following are two important cases which have been under my special inspection and treatment for some time. The first, a Mr. E. Short, of Saginaw, W. S., aged about seventy. The trouble came on about six years ago while living in Texas. It began by a slight twitching of the muscles under the right eye. There was no pain at any time. One year from the time it started he consulted a dentist who extracted the right upper cuspid, but this gave him no relief. Now all the upper teeth are wanting, but the trouble still exists, being periodical in its nature, and the right eye partly closed.

Another was that of a lady, aged about forty. Her trouble came on about four years ago, and in very much the same manner as that of the previous one. She has a twitching of the left masseter muscle at intervals, and also of the lower eyelid of the corresponding side. This trouble has been a source of a great deal of annoyance to her, so much so that she consulted a number of physicians with the hope of getting relief. She was finally induced to have her teeth examined. I extracted all the diseased teeth that she had in her mouth some time ago, and instructed her to report to me in case she received any benefit therefrom; but I have thus far been unable to get any word from her.

There are many well marked cases on record in which the cause of this muscular disturbance of the face was found to be due to an irritation reflected from diseased teeth.

The subject is of the profoundest interest and importance, but is little understood even by our best investigators. One fact, however, seems to be apparent, and that is that there is some abnormal relation between efferent and afferent nerve impulses. The trigeminal nerve is undoubtedly the most remarkable nerve in the whole nervous system, especially when we consider the intricate connection of its nuclei with those of the various motor cranial nerves.

This will undoubtedly afford ample explanation for the many reflex disturbances. But why this irritation due to pathological conditions of the teeth, for example, should in one result in a wry neck or facial spasm, and in another in blindness, in deafness, in chorea, in dyspepsia, in epilepsy or in mania, is an inquiry at present unanswerable and almost beyond our comprehension.

As "habit spasms," or whatever muscular disturbance it may be, are generally increared by observation, it is very important that little notice should be taken of them by the friends of the patient. Sometimes the movements will then cease without further treatment.

They are seldom under direct voluntary control, and the endeavor to prevent their occurrence may be futile, especially if the attempt is made under the fear of threat and punishment. But the promise of a reward at the close of each day on which the spasm has not occurred will sometimes gradually cause their disappearance; a strong desire, free from any depressing emotion, effects that which the will cannot directly achieve. Any obvious defect in the health must be made good, and a change of air is often very beneficial, especially when a change in companionship can be secured at the same time. The deterring influence of strangers is often very marked. Nerve tonics, such as quinin, are sometimes of use. If there is much excitability of the brain, or if spasmodic movements are seen, bromid of potassium may be needed, and occasionally a local blister is very efficient. The smiling clergyman mentioned above ceased to be troubled after he had for a few weeks taken some iodid of iron, and a dose of bromid each time he had to conduct a service in church.

In recent cases of facial spasm, apparently excited by cold, free diaphoresis should be employed, and the face and side of the head bathed frequently with hot water. If there are indications of organic disease, the nature of this must be ascertained, and, as far as possible, treated. All causes of reflex irritation must be sought for and removed; decayed teeth should be extracted, especially if they are on the same side as the spasm. Occasionally such nervine tonics and stimulants as zinc, nitrate of silver, asafœtida and valerian may be given, but in the vast majority of cases they conspicuously fall. Sedatives applied to the skin seldom exert any influence over the spasm. In the use of morphia for the relief of spasm it is probably more desirable to inject it in or near the seat of spasm than in its anodyne use. The temple is the most convenient locality. Electricity has been largely used, and has been highly praised by some of its advocates, but in nine-tenths of the cases it fails even to relieve.

Stretching the facial nerve has been of late adopted, but with small results. However trivial many of these cases seem to be, they often cause more annoyance than many diseases of far more serious nature.—*Items of Interest.*

---

